# DNA methylation and expression of LINE-1 and HERV-K provirus sequences in urothelial and renal cell carcinomas

**DOI:** 10.1038/sj.bjc.6690524

**Published:** 1999-07

**Authors:** A R Florl, R Löwer, B J Schmitz-Dräger, W A Schulz

**Affiliations:** Urologische Klinik, Center for Biological and Medical Research, Heinrich-Heine-Universität, Moorenstraße 5, Düsseldorf, D-40225; Paul-Ehrlich-Institut, Paul-Ehrlich-Str. 51, Langen, D-63225, Germany

**Keywords:** bladder cancer, renal cancer, retrotransposon, methylcytosine, chromosome instability

## Abstract

Since DNA methylation is considered an important mechanism for silencing of retroelements in the mammalian genome, hypomethylation in human tumours may lead to their reactivation. The methylation status of LINE-1 retroposons was determined in 73 samples of urinary bladder cancers, 34 specimens of renal cell carcinoma and in the corresponding normal tissues by Southern blot analysis. LINE-1 sequences were strongly methylated in normal tissues and were significantly hypomethylated in 69 (95%) urothelial carcinomas, but in none of the renal carcinomas. Hypomethylation in bladder cancers was independent of stage and tended to increase with grade. The methylation status of HERV-K proviral DNA in normal and transformed urothelial cells paralleled that of LINE-1 sequences (*r*^2^ = 0.87). It was shown by ligation-mediated polymerase chain reaction that hypomethylation also extended to the LINE-1 promoter sequence located at the 5′-ends of full-length elements which is repressed by methylation in somatic tissues. Accordingly, full-length LINE-1 transcripts were detected by Northern blot analysis in two urothelial carcinoma cell lines. In contrast, transcripts from HERV-K proviruses were restricted to teratocarcinoma cell lines. Our data indicate that genome-wide DNA hypomethylation is an early change in urothelial carcinoma, but is absent from renal cell carcinoma. The coordinate changes of LINE-1 and HERV-K DNA methylation suggest that hypomethylation in urothelial cancer affects a variety of different retroelements to similar extents. We speculate that decreased methylation of LINE-1 retroelements, in particular, may contribute to genomic instability in specific human tumours such as urothelial carcinoma by rendering these normally repressed sequences competent for transcription and recombination. © 1999 Cancer Research Campaign


*British Journal of Cancer* (1999) **80**(9), 1312–1321
© 1999 Cancer Research Campaign
Article no. bjoc.1999.0524

### DNA methylation and expression of LINE-1 and HERV-K provirus sequences in urothelial and renal cell carcinomas

**AR Florl[1], R Löwer[3], BJ Schmitz-Dräger[1] and WA Schulz[1,2]**

[1]Urologische Klinik and [2]Center for Biological and Medical Research, Heinrich-Heine-Universität, Moorenstraße 5, D-40225 Düsseldorf, [3]Paul-Ehrlich-Institut, Paul-Ehrlich-Str. 51, D-63225 Langen, Germany

**Summary** Since DNA methylation is considered an important mechanism for silencing of retroelements in the mammalian genome, hypomethylation in human tumours may lead to their reactivation. The methylation status of LINE-1 retroposons was determined in 73 samples of urinary bladder cancers, 34 specimens of renal cell carcinoma and in the corresponding normal tissues by Southern blot analysis. LINE-1 sequences were strongly methylated in normal tissues and were significantly hypomethylated in 69 (95%) urothelial carcinomas, but in none of the renal carcinomas. Hypomethylation in bladder cancers was independent of stage and tended to increase with grade. The methylation status of HERV-K proviral DNA in normal and transformed urothelial cells paralleled that of LINE-1 sequences ($r^2 = 0.87$). It was shown by ligation-mediated polymerase chain reaction that hypomethylation also extended to the LINE-1 promoter sequence located at the 5′-ends of full-length elements which is repressed by methylation in somatic tissues. Accordingly, full-length LINE-1 transcripts were detected by Northern blot analysis in two urothelial carcinoma cell lines. In contrast, transcripts from HERV-K proviruses were restricted to teratocarcinoma cell lines. Our data indicate that genome-wide DNA hypomethylation is an early change in urothelial carcinoma, but is absent from renal cell carcinoma. The coordinate changes of LINE-1 and HERV-K DNA methylation suggest that hypomethylation in urothelial cancer affects a variety of different retroelements to similar extents. We speculate that decreased methylation of LINE-1 retroelements, in particular, may contribute to genomic instability in specific human tumours such as urothelial carcinoma by rendering these normally repressed sequences competent for transcription and recombination.

**Keywords:** bladder cancer; renal cancer; retrotransposon; methylcytosine; chromosome instability

In human somatic tissues between 70 and 80% of all cytosine bases in the sequence CpG are modified in a tissue-specific pattern by addition of a methyl group at the 5′ position of the pyrimidine base. Two kinds of alterations in DNA methylation patterns, hypermethylation and hypomethylation, respectively, have been observed in human tumours (Laird et al, 1996; Baylin et al, 1998; Schulz, 1998). Hypermethylation denotes the increased methylation of CpG-rich gene promoter sequences restricted to specific genes or genomic regions. Hypermethylation inactivates, for instance, the VHL tumour suppresser gene in some renal cell carcinomas (Herman et al, 1994; Clifford et al, 1998) and the MTS1/p16INK4 tumour suppresser gene in some urothelial, prostate and other carcinomas (Gonzalez-Zulueta et al, 1995; Herman et al, 1995; Merlo et al, 1995; Jarrard et al, 1997). In contrast, hypomethylation appears to affect the genome at large and often results in a decreased overall content of methylcytosine. However, it is not clear which sequences exactly are subject to hypomethylation. DNA hypomethylation occurs as an early change in some kinds of carcinomas, but is associated with progression in others, such as prostate carcinoma (Bedford et al, 1987; Santourlidis et al, 1998). A study of 13 bladder cancers showed a high prevalence of DNA hypomethylation, but due to the low number of samples the relationship towards tumour stage and grade could not be elucidated (Jürgens et al, 1996). A systematic study in renal cell carcinoma has not been reported.

*Received 2 November 1998*
*Revised 21 November 1998*
*Accepted 27 January 1999*

*Correspondence to:* WA Schulz

Genome-wide changes in DNA methylation may, in particular, affect those repetitive DNA sequences that are comparatively rich in CpG dinucleotides such as Alu, other SINE, L1 LINE (LINE-1) and certain satellite sequences that between them contain a considerable fraction of total methylcytosine in the human genome (Miller et al, 1974). Since DNA methylation also limits the ability of retroelements to be activated and transcribed and to participate in recombination, methylation of retrotransposons and proviruses probably also counteracts their tendency to endanger the stability of the genome by amplification and recombination (Yoder et al, 1997).

Two very distantly related repetitive retroelements were investigated in this study. LINE-1 retrotransposons constitute up to 15% of the human genome (Kazazian and Moran, 1998). Complete elements are 6-kb long containing a CpG-rich internal promoter located at their 5′-ends and two open reading frames that encode an RNA-binding protein and an endonuclease/reverse transcriptase (Feng et al, 1996). Most LINE-1 elements are defective due to truncations at their 5′-end or internal mutations. Their total number exceeds $10^5$, among which approximately 100 have remained transposition-competent (Moran et al, 1996). Disruption of genes by insertion of LINE-1 elements has been found in human cancer and genetic disease (Morse et al, 1988; Miki et al, 1992; Dombroski et al, 1993; Holmes et al, 1994). In addition,

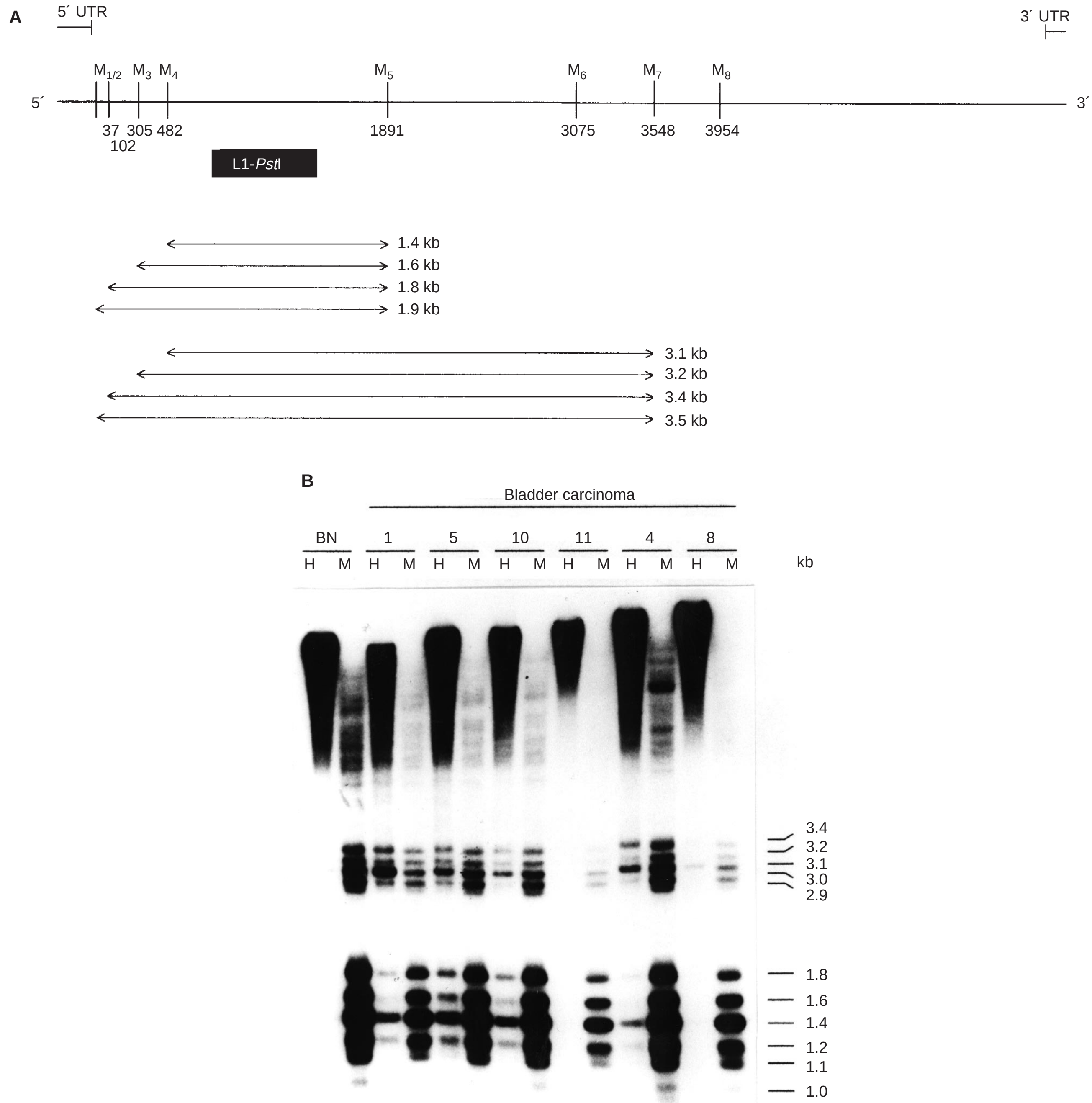

**Figure 1** Southern blot analysis of LINE-1 methylation in urothelial cancer. (**A**) Restriction map of LINE-1 elements. Top: restriction map of the cloned element L1.2B with positions of the *Msp*I/*Hpa*II sites. The location of the *Pst*I fragment used as a probe is indicated. Below: the two groups of double-headed arrows denote the series of bands resulting from successive mutation or methylation of sites M4 to M1 with or without mutation or methylation of sites M5 and M6 (top vs bottom). (**B**) LINE-1 methylation in urothelial carcinoma. Analysis of LINE-1 methylation in six samples of urothelial carcinomas (nos 1, 5, 10, 11, 4, 8). BN: normal bladder. The sizes of the observed bands are indicated on the right. H: *Hpa*II digestion; M: *Msp*I digestion. Samples 1, 5 and 10 show moderate hypomethylation, sample 4 weak hypomethylation, and samples 8 and 11 no hypomethylation

LINE-1 sequences have been identified at or near chromosomal translocation sites (Nagarajan et al, 1990; Pomykala et al, 1994; Liu et al, 1997). Presumably to prevent such accidents, most elements are highly methylated in normal adult tissues (Alves et al, 1996; Jürgens et al, 1996; Woodcock et al, 1997). Methylation of the LINE-1 promoter sequence has been shown to repress its activity (Hata et al, 1996).

The human endogenous retrovirus type K (HERV-K) family comprises 30–50 full-length members per haploid genome, among which several contain intact open reading frames (reviewed in Löwer et al, 1996; Tönjes et al, 1996). Intact elements are flanked by two long terminal repeats each approximately 1-kb in length providing the promoter and poly-adenylation signals respectively. HERV-K are the most intact among human endogenous retroviruses

*© 1999 Cancer Research Campaign*

**Table 1** LINE-1 hypomethylation and tumour grade and stage in urothelial carcinoma

| | | Hypomethylation (no. of cases) | | | | |
|---|---|---|---|---|---|---|
| **Tumour grade** | **None** | **2–10%** | **11–20%** | **20–30%** | **> 30%** | **Total** |
| G1 | 0 | 1 | 0 | 0 | 0 | 1 |
| G2 | 2 | 9 | 5 | 5 | 3 | 24 |
| G3 | 2 | 19 | 8 | 7 | 7 | 43 |
| G4 | 0 | 0 | 3 | 0 | 2[a] | 5 |
| Total | 4 | 29 | 16 | 12 | 12 | 73[b] |
| **Tumour stage** | **None** | **2–10%** | **11–20%** | **20–30%** | **> 30%** | **Total** |
| pTa | 1 | 2 | 1 | 2 | 2 | 8 |
| pT1 | 0 | 4 | 2 | 1 | 3 | 10 |
| pT2 | 2 | 6 | 1 | 2 | 1 | 12 |
| pT3 | 1 | 12 | 8 | 6 | 4 | 31 |
| pT4 | 0 | 5 | 4 | 1 | 1 | 11 |
| pTis | 0 | 0 | 0 | 0 | 1 | 1 |
| Total | 4 | 29 | 16 | 12 | 12 | 73[b] |

[a]Including one large area carcinoma in situ. [b]Including the 13 tumour samples published in Jürgens et al, 1996.

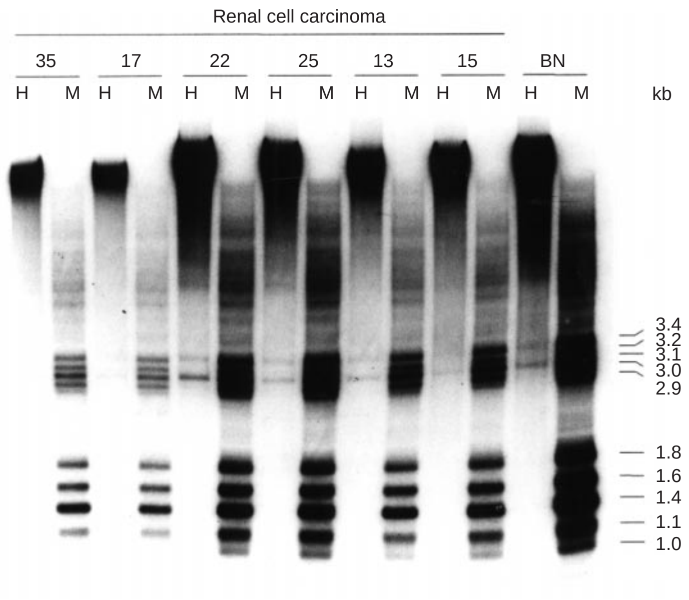

**Figure 2** Southern blot analysis of LINE-1 methylation in renal carcinoma. Analysis of LINE-1 methylation in six samples of renal cell carcinoma (nos 35, 17, 22, 25, 13, 15). BN: normal bladder. KN: normal kidney. The sizes of the observed bands are indicated on the right (cf. Figure 1A). H: *Hpa*II digestion; M: *Msp*I digestion and make up 1–2% of the human genome. HERV-K sequences are relatively poor in CpG dinucleotides, for instance there are fewer than 20 in their long terminal repeats. Nevertheless, methylation of these sequences like that of LINE-1 sequences may be important in controlling their ability to be transcribed, to retrotranspose and to participate in recombination. For instance, LINE-1 (Bratthauer and Fanning, 1992) and HERV-K (Löwer et al, 1995; Gotzinger et al, 1996) sequences have been shown to be hypomethylated and transcribed in human teratocarcinoma cells.

In the present study, methylation of LINE-1 elements was investigated in a large number of urothelial carcinoma and renal cell carcinoma specimens to determine the relationship of hypomethylation to tumour stage and tumour grade. To determine its specificity, hypomethylation of HERV-K sequences was also investigated. To elucidate whether hypomethylated retroelements might become reactivated in tumour cells, methylation of the LINE-1 promoter at their 5′-end was investigated by a newly adapted ligation-mediated (LM-) polymerase chain reaction (PCR) method and the expression of LINE-1 and HERV-K sequences was compared in bladder carcinoma and teratocarcinoma cell lines.

### MATERIALS AND METHODS

### Cell lines

Human bladder carcinoma cell lines (Grimm et al, 1995), male teratocarcinoma Tera-1, NCCIT, and GH, female teratocarcinoma Pa 1, amnion AV3, choriocarcinoma JAR, Molt 4 T-cell lymphoma and rhabdomyosarcoma A204 cell lines were cultured as described (Löwer et al, 1993).

 *© 1999 Cancer Research Campaign*

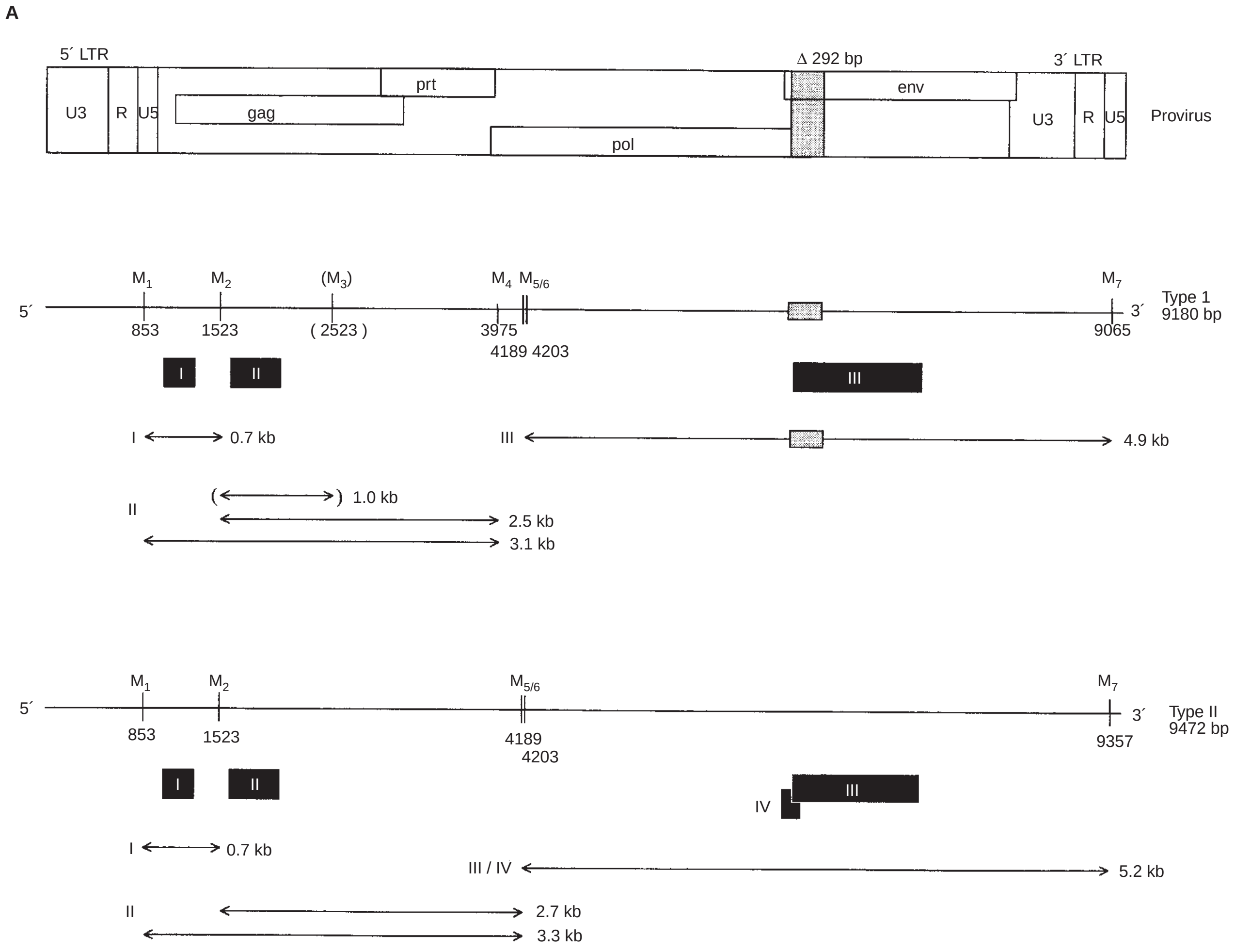

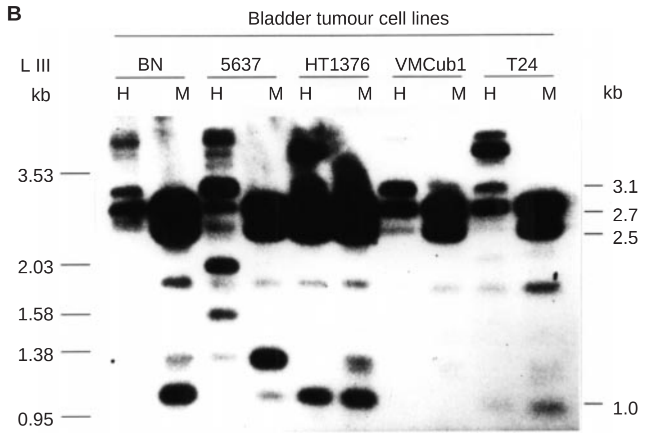

**Figure 3** Southern blot analysis of HERV-K methylation in bladder carcinoma cell lines. (**A**) Restriction map of HERV-K proviruses. *Msp*I partial restriction maps of a cloned provirus are aligned with gene maps of HERV-K proviruses. Type I and II proviruses are distinguished essentially by the indicated 292-bp deletion (light grey boxes). Dark boxes below the restriction map labelled I–IV indicate the locations of the hybridization probes used and the groups of double-headed arrows labelled in the same fashion at the bottom of the drawing show the main bands expected upon Southern analysis. (**B**) Southern blot analysis. DNA from normal bladder (BN) or from the indicated bladder carcinoma cell lines was digested with *Hpa*II (H) or *Msp*I (M), separated, blotted and hybridized with probe II. DNA marker sizes are indicated on the left side and some of the identified bands (cf. panel **A**) on the right side of the blot

### Patients and tissue samples

Bladder and renal tissue samples were obtained by cystectomy or nephroureterectomy. Tumours and corresponding normal tissues were dissected immediately after surgery, 0.1–0.5 g blocks were frozen in liquid nitrogen and stored at –80°C. In the present study, 60 samples of urothelial carcinoma were investigated in addition to the 13 samples investigated previously (Jürgens et al, 1996). Among the 73 patients, 53 were male and 20 were female. Their mean age was 67 years with a range from 41 to 85 years. In all, eight tumours were staged as pTa, ten carcinomas as pT1 and 12 as pT2, 31 as pT3 and 11 as pT4. One tumour was graded as G1, 24 tumours were graded as G2, 43 as G3 and four as G4. One patient

© 1999 Cancer Research Campaign had a large carcinoma in situ. Among the 34 patients with renal cell carcinoma, 22 were male and 12 were female. Their mean age was 63 years with a range from 42 to 81 years. One renal carcinoma was staged as pT1, 23 as pT2, nine as pT3 and one as pT4. One sample was graded G1 and eight G3, all others were classified as G2. Six renal carcinomas showed chromophilic histology, the remainder were clear cell carcinoma. Grading and staging were performed according to the 1992 UICC classification for bladder carcinoma and according to the Thoenen classification for renal cell carcinoma. This study was approved by the Ethics Committee of the Heinrich-Heine-University.

### DNA extraction and Southern blot analysis

DNA extraction was performed as described (Jürgens et al, 1996). Southern blot analysis was performed using a 0.6-kb *Pst*I fragment from the cloned (Dombroski et al, 1993) LINE element pL1.2 B (kindly provided by Dr Kazazian, Baltimore, MD, USA) or fragments from cloned HERV-K proviruses (Löwer et al, 1995) as detailed in Figures 1A and 3A. The extent of hypomethylation was quantified by video densitometry as follows. Defined bands in the 1.0–4.0 kb range in the case of LINE-1 elements and the 2.5–3.1 kb bands resulting from hybridization of HER V-K proviruses with probe II, were quantified by Gaussian approximation and the sum of the band intensities in the *Hpa*II lane was divided by the sum of the band intensities in the *Msp*I lane from the same sample. This value was corrected for unequal loading of the two lanes which yielded a factor of less than 1.5 in all cases and the hypomethylation background of normal bladder or kidney.

### LM-PCR

The method was modified from (Steigerwald et al, 1990). Usually 1 μg of DNA was digested with 10 U *Hpa*II or *Msp*I for 4 h. The linker primer (5′-GCGGTGACCCGGGAGATCTGAATTC-3′) and linker 2 (5′-CGGAATTCAGATC-3′) were annealed, and 15 pmole of the product were ligated to 1 μg of digested DNA. Following removal of the ligase, 300 ng of DNA were amplified using 12 pmol each of linker primer and either primer F2 (5′-TTTTGTTTGTCTGTGCCCTGCCCCC-3′) or primer F3 (5′-ACCTAAGCAAGCCTGGGCAATG-3′), 0.25 mmol $l^{-1}$ of each deoxynucleotide triphosphate, 1.3 U *Taq* polymerase, 1.5 mmol $l^{-1}$ magnesium chloride, 50 mmol $l^{-1}$ potassium chloride, 100 mmol $l^{-1}$ Tris, pH 8.3, in a total volume of 50 μl for 13 cycles. The annealing temperature was 67°C and the extension temperature 73°C. Ten microlitres from each reaction were loaded onto a 2.5% agarose gel, electrophoresed and blotted to a nylon membrane. The membrane was probed with linker primer tailed with digoxigenin–ddUTP using a 3′-tailing kit (Boehringer Mannheim, Germany) at 65°C in 5 × standard saline citrate (SSC), 0.1% lauroyl sarcosine, 0.02% sodium dodecyl sulphate (SDS) and 1% blocking reagent and washed with 2 × SSC, 0.1% SDS and 0.1 × SSC, 0.1% SDS twice each for 5 min at 65°C. The labelled probe was detected by luminescence as described by the supplier (Boehringer Mannheim, Germany). In vitro methylation of DNA with *Hpa*II methylase (New England Biolabs, Bad Homburg, Germany) was performed as suggested by the supplier.

### RNA extraction and Northern blot analysis

Preparation of polyA-selected mRNA and Northern analysis were performed as described (Löwer et al, 1995). Probes used for hybridization comprised a 3.4 *Eco*RI fragment representing the 5′-part of the LINE-1 cDNA clone cD11 A (Skowronski et al, 1988) which was a kind gift of Dr M Singer (Baltimore), a 165 bp *Bam*HI/*Eco*RI fragment from the R/U5 region of a HTDV/HERV-K provirus, and a 1.8 kb *Pst*I fragment from chicken β-actin (Löwer et al, 1995).

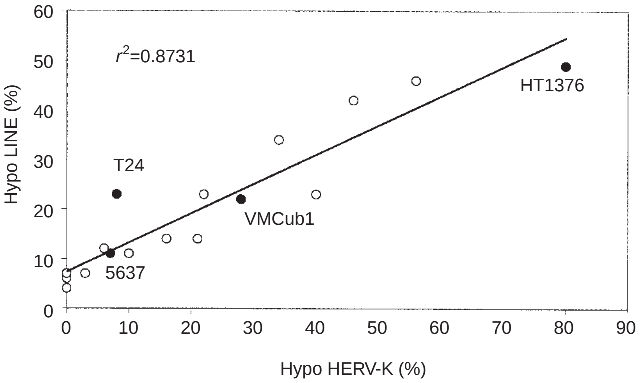

**Figure 4** Hypomethylation of LINE-1 and HERV-K sequences in urothelial carcinoma. Hypomethylation of LINE-1 and HERV-K sequences as quantified by video densitometry of Southern blots using the *Pst*I probe and probe II, respectively (cf. Figure 1A and 3A), are plotted against each other for 13 urothelial carcinoma samples (open circles) and four cell lines (filled circles)

### RESULTS

### LINE-1 methylation in urothelial tumours

Figure 1 shows the results of a typical Southern blot analysis of LINE-1 methylation using a 0.6-kb *Pst*I fragment from the cloned element L1.2B (Dombroski et al, 1993) as a probe. L1.2B contains eight sites for the restriction enzymes *Msp*I and *Hpa*II numbered M1–M8 (Figure 1A). Both enzymes recognize the sequence CCGG, but *Hpa*II is inhibited by methylation of either internal cytosine, whereas *Msp*I is only inhibited by methylation of an outer cytosine, which is uncommon in mammalian cells. L1.2B is expected to yield upon Southern hybridization a 1.4-kb band following restriction with *Msp*I. Due to the polymorphy of LINE-1 elements, several bands are observed (Figure 1). Successive loss of the restriction sites M4–M2 yields bands of 1.6 kb, 1.8 kb and 1.9 kb respectively. About half of all LINE-1 elements hybridizing to the probe lack sites M5 and M6 producing a further series of bands from 3.1 to 3.5 kb. Some of these bands could theoretically result from elements lacking site M5 only (cf. Figure 1A). However, since cloned sequences usually lack both sites, we presume that most bands stem from such elements. Further recognition sites for the enzyme pair present in individual elements result in additional bands, most conspicuously at 1.1 and 1.2 kb.

As expected, LINE-1 DNA sequences were strongly methylated in normal bladder. This is illustrated by the almost complete lack of specific bands in the smaller than 5 kb range upon *Hpa*II digestion of DNA isolated from normal tissue (Figure 1B). In contrast, almost all samples of urothelial carcinoma displayed specific hybridization signals in this size range indicating hypomethylation of LINE-1 elements compared to normal tissue (Figure 1B). Some samples showed methylation of LINE-1 elements slightly diminished compared to normal bladder tissue (specimen no. 8 and no. 11 in Figure 1B), whereas others showed extensive hypomethylation (specimen no. 1 and no. 5 in Figure 1B). However, some

 © 1999 Cancer Research Campaign

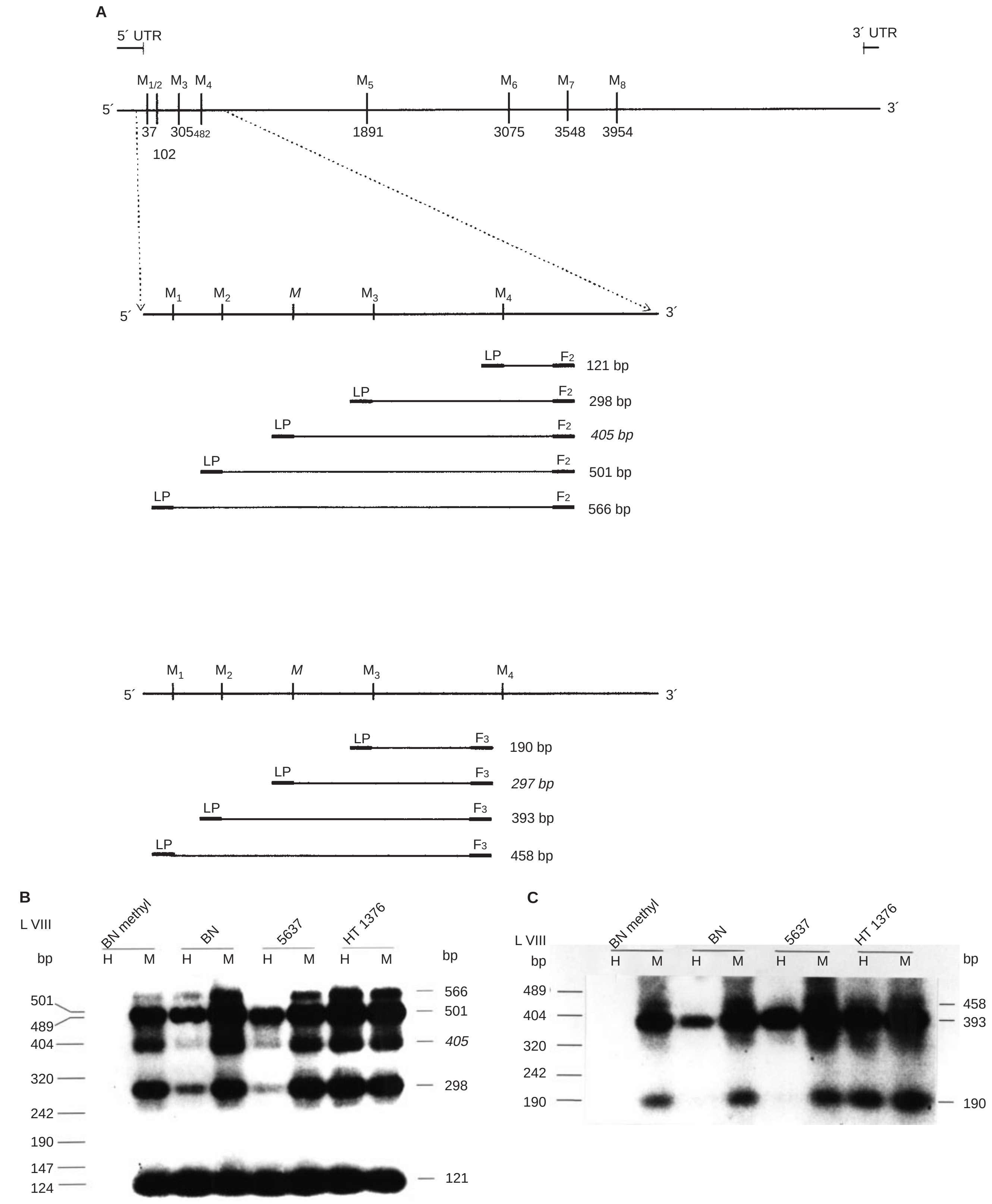

**Figure 5** Ligation-mediated PCR analysis of LINE-1 promoter methylation. (**A**) LM-PCR products expected from hypomethylated LINE-1 sequences. The two panels show the series of products expected after ligation-mediated PCR using primers F2 (upper panel) or F3 (lower panel). The positions of the *Msp*I/*Hpa*II sites (M1–M8) are indicated. The additional *Msp*I site discovered during this study is indicated by an italicized M. Note that all products contain the linker primer. (**B, C**) LINE-1 hypomethylation in bladder tissue and urothelial carcinoma cell lines. Results of LM-PCR reaction using primers F2 (**B**) and F3 (**C**) respectively. The four DNA samples investigated were from normal bladder (BN) or the bladder carcinoma cell lines 5637 and HT1376. The BN DNA on the left (labelled ‘methyl’) was methylated with *Hpa*II methylase prior to treatment with restriction enzymes. The sizes of the DNA marker used are indicated on the left and the probable sizes of the products on the right. H: *Hpa*II digestion; M: *Msp*I digestion

*© 1999 Cancer Research Campaign*

degree of hypomethylation was found in almost all (69/73 = 95%) urothelial carcinomas. Hypomethylation was not related to tumour stage (Table 1). For instance, the extent of hypomethylation ranged from 0 to 60% in papillary tumours (pTa) and from 5 to 37% in carcinomas invading neighbouring tissues (pT4). The extent of hypomethylation tended to increase with tumour grade (Table 1), but this relationship was not significant (Mantel–Haenszel $\chi^2$ exact test: $P = 0.127$; asymptotic Fisher's test: $P = 0.070$).

### Methylation of LINE-1 sequences in renal cell carcinoma

In contrast to urothelial carcinomas, hypomethylation of LINE-1 sequences was not observed in any of 34 renal cell carcinoma specimens from various stages and grades. A typical Southern blot analysis is shown in Figure 2.

### HERV-K methylation in urothelial carcinoma

Figure 3A displays the structure (top), restriction maps (centre) of prototypic type I and type II HERV-K along with the location of the probes I–IV used for Southern hybridization and major fragments expected and found. Type I and II HERV-K proviruses differ essentially by a 292-bp deletion at the amino-terminus of the env gene also removing the coding region of the c-orf gene. The recognition sites of *Msp*I and *Hpa*II are labelled M1–M7 according to their location in the cloned type I proviruses HERV-K10. Note that site M3 occurs only in this particular provirus.

Methylation of the HERV-K provirus DNA sequences was first compared in urothelial carcinoma cell lines with different degrees of LINE-1 hypomethylation (Figure 3B). Following digestion with *Msp*I, probe II located within the gag region between sites M2 and M3 yielded mainly two bands of approximately 2.5 kb and 2.7 kb. The 1.0 kb band resulting from HERV-K10 was also observed in normal bladder and in two cell lines, but was absent from VMCub1 and weaker than expected in T24, suggesting loss of this particular provirus in these cell lines (Figure 3A). The double band at 2.5 kb and 2.7 kb suggests that approximately half of the HERV-K proviruses lack site M4. Since cloned type I proviruses, but not type II proviruses, contain this site (unpublished observations) the double band reflects essentially the two classes of proviruses (cf. Figure 3A). In addition to the major bands, a few weaker additional bands were observed in the *Msp*I digest that may be derived from truncated or internally deleted elements. Very little signal was detected in the lower molecular weight range in the *Hpa*II lane from normal bladder indicating methylation of probably both sites flanking the probe. Methylation was significantly diminished in all bladder carcinoma cells with HT1376 DNA almost devoid of methylation. In the other cell lines a 3.1 kb band was prominent in the *Hpa*II lanes that may correspond to the M1–M4 fragment resulting from selective methylation of site M2. The methylation pattern in cell line 5637 most closely resembled that in normal mucosa, whereas the other two cell lines showed intermediate levels of methylation. This order of degree of methylation was also seen with the other HERV-K probes indicated in Figure 3A (data not shown).

HERV-K methylation paralleled LINE-1 methylation in the cell lines as well as in 13 primary tumours representing various degrees of LINE-1 hypomethylation (Figure 4, $r^2 = 0.87$). The main difference was that a low level of LINE-1 hypomethylation was measured in some tumours without detectable HERV-K hypomethylation.

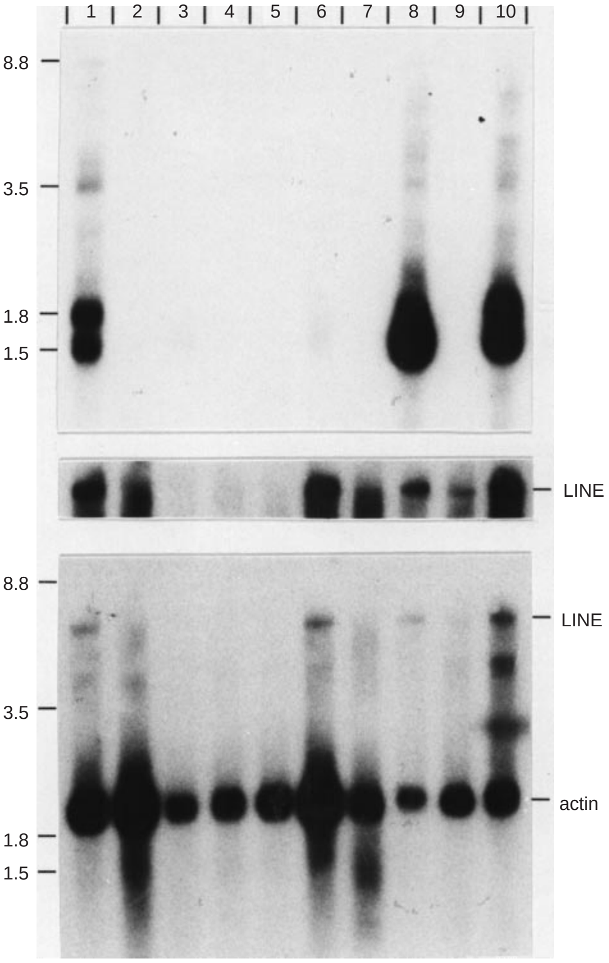

**Figure 6** Northern blot analysis of LINE-1 and HERV-K expression in bladder carcinoma cell lines. Two micrograms mRNA from the cell lines Tera 1 (lane 1), Molt 4 (lane 2), AV3 (lane 3), JAR (lane 4), A 204 (lane 5), Pa 1 (lane 6), 5637 (lane 7) NCCIT (lane 8), HT1376 (lane 9) and GH (lane 10) were separated on a denaturing agarose gel, blotted and hybridized with probes against HERV-K (top) or LINE-1 and β-actin (bottom). A slightly longer exposure of the LINE signal is shown in the centre. The sizes of the HERV-K mRNAs are indicated on the left in kb and the locations of the transcripts for LINE-1 and actin are marked on the right

### LM-PCR analysis of LINE-1 methylation

To analyse the methylation status of the LINE-1 promoter region, an LM-PCR method was used. A staggered linker was ligated to the CG overhang resulting from DNA restriction by either *Hpa*II or *Msp*I, and the ligation products were amplified by PCR using a linker-primer (LP) and an LINE-1-specific primer. The reaction products were separated on an agarose gel, blotted and visualized by hybridization with digoxigenin–ddU-tailed linker primer. Since specific bands are only produced if a restriction enzyme has cut, this method yields a positive display of hypomethylated sites in the *Hpa*II lane. The target-specific primers F2 and F3 (Figure 5A) were chosen from the sequence of the active element L.1.2B which upstream of the primers contains four (F2) or three (F3) recognition sites, respectively, for the restriction enzymes (Figure 5A), corresponding to sites M1–M4 in Figure 1A. Since LINE-1

© 1999 Cancer Research Campaign elements are polymorphic, several PCR products are expected following *Msp*I digestion (Figure 5A). All predicted products were observed in the *Msp*I lane (Figure 5B). An additional band around 400 bp is probably accounted for by LINE-1 elements, e.g. in the human β-globin locus (EMBL accession number X03145), containing an additional *Msp*I site at bp 405 while lacking sites M3 and M4.

A comparison of LINE-1 5′-end methylation in normal bladder and the cell lines 5637 and HT1376 revealed that their difference in overall LINE-1 methylation shown previously (Jürgens et al, 1996) by Southern analysis also extended to the 5′-ends (Figure 5 B, C). Whereas the bands in the *Hpa*II lanes from normal bladder and from the cell line 5637 differed in intensity from those in the *Msp*I lanes, almost identical patterns were observed with HT1376 DNA. However, residual bands were seen in DNA from normal bladder in the *Hpa*II lane. The F2 primer yielded predominantly the 121-bp and with lower intensity the 501-bp band (Figure 5B). The same pattern of bands was also observed in five additional DNA samples from normal bladder, in normal prostate and in normal kidney DNA (data not shown). The similar intensity of the 121-bp band in the *Hpa*II and *Msp*I lanes indicates that site M4 is rarely methylated in those LINE-1 elements annealing with primer F2. Since no increased intensity was seen in any of the bands originating from sites M1–M3 with the upstream F3 primer (Figure 5C), these sites are probably methylated in most of these elements in normal tissue. With primer F3, the 297-bp band corresponding to the 405-bp band upon use of primer F2 did not appear. Indeed, the LINE-1 elements from the β-globin locus contain several base changes in the region corresponding to primer F3 compared to LINE-1.2B. Methylation of DNA from normal bladder with *Hpa*II methylase in vitro prior to enzyme digestion and LM-PCR did not alter the band pattern in the *Msp*I lanes, but caused a complete disappearance of the bands in the *Hpa*II lanes (Figure 5 B, C) confirming their origin from unmethylated sites.

In contrast to the 121-bp band, the intensities of all other bands increased in the *Hpa*II lanes from most bladder carcinoma cell lines and primary tumour samples compared to normal bladder (Figure 5 B, C). For instance, the 501-bp band whose presence indicates hypomethylation of site M2 in some LINE-1 elements was much weaker in the *Hpa*II lane from normal bladder than in HT1376 DNA, and its intensity increased in many tumour samples. Eight tumour samples investigated by both Southern blot analysis and by LM-PCR gave concordant results with both methods (data not shown) indicating that hypomethylation of LINE-1 sequences in urothelial carcinoma also extends to their 5′-ends containing the promoter sequence.

### Expression of retroelements in carcinoma cell lines

To investigate the expression of LINE-1 and HERV-K mRNAs, a Northern blot containing polyA-selected RNA from the bladder carcinoma cell lines 5637 and HT1376, several teratocarcinoma cell lines and others from different tissue origin was successively hybridized with probes for HERV-K, LINE-1 and β-actin (Figure 6). The HERV-K probe detected transcripts exclusively in the three male teratocarcinoma cell lines Tera 1, NCCIT and GH (lanes 1, 8 and 10 in Figure 6). The 1.5-kb mRNA with unknown coding function and the 1.8-kb c-orf mRNA were very strongly expressed in all three cell lines, whereas the 3.5-kb env mRNA and the 8.8-kb genomic RNA were present at much lower levels. None of the other cells showed a comparable level of transcription. Expression of 6-kb LINE-1 transcripts was also most prominent in the teratocarcinoma cell lines, but these transcripts were also observed (in decreasing order of expression levels) in the female teratocarcinoma, in the bladder carcinoma cell lines HT1376 and 5637, and in the T-lymphoma cell line Molt 4.

### DISCUSSION

The present study reveals substantial differences in DNA methylation between urothelial and renal cell carcinoma. In urothelial carcinoma, in accord with our initial report (Jürgens et al, 1996) hypomethylation of LINE-1 sequences was highly prevalent. The substantial number of tumour specimens investigated in the two studies allows the conclusion that hypomethylation is not related to tumour stage but that higher grade tumours tend to more pronounced hypomethylation. High prevalence throughout all tumour stages and grades is the hallmark of an early change. Therefore these data suggest that hypomethylation of LINE-1 sequences is an early event during the development of urothelial cancer. Since the number of low-grade and low-stage tumours in our study was small, a more extensive study of early stage urothelial tumours and their precursors is needed to definitely answer this issue. Urothelial carcinoma in this respect may resemble colon cancer (Goelz et al, 1985; Feinberg et al, 1988), but obviously differs from prostate carcinoma where hypomethylation seems to be associated with tumour progression (Bedford et al, 1987; Santourlidis et al, 1999). Renal cell carcinoma obviously belongs to a distinct class of tumours which lack DNA hypomethylation altogether. Interestingly, hypermethylation of CpG-islands in the promoter regions of tumour suppressor genes has been described in renal cell carcinoma as well (Makos et al, 1993; Herman et al, 1995; Clifford et al, 1998). Thus, hypermethylation appears to occur in renal cell carcinoma independently from hypomethylation suggesting that these alterations in DNA methylation are not necessarily linked to each other. Since hypermethylation of a crucial tumour suppresser gene will provide a selective advantage for the affected tumour cell, it is conceivable that incidental alterations of DNA methylation in crucial genes such as the von-Hippel–Lindau gene are selected for in renal cell carcinoma, whereas the multiple alterations of DNA methylation patterns in urothelial carcinoma may be due to overall deregulation of DNA methylation.

The relative methylation of LINE-1 retrotransposons and of HERV-K proviruses were highly significantly correlated in urothelial carcinoma cell lines as well as in primary tumours (Figure 4). Although both classes of sequences belong to the general category of retroelements, they are very distantly related and differ strongly in their overall content and distribution of methylation target sites. Therefore, DNA hypomethylation in urothelial carcinoma involves quite different sequences to a similar extent and can truly be designated ‘genome-wide’. Conversely, this finding confirms that methylation of LINE-1 sequences can serve as a good marker of changes in overall DNA methylation in human tumours.

Deregulation of DNA methylation is thought to contribute to genomic instability in human tumours, although the mechanisms involved are not understood. Reactivation of retroelements might represent a particularly important consequence of DNA hypomethylation, since their containment is a key function of DNA methylation in mammalian genomes (Yoder et al, 1997). Retrotransposition of LINE-1 sequences results in a highly unstable branched DNA structure prone to undergoing recombina-

© 1999 Cancer Research Campaign tion with accessible elements located nearby or even elsewhere in the genome (Feng et al, 1996). Chromosome deletions and translocations probably caused by retrotransposition events have indeed been observed in several human cancers (Morse et al, 1988; Nagarajan et al, 1990; Miki et al, 1992; Pomykala et al, 1994; Liu et al, 1997). The decrease in promoter methylation in urothelial tumour cells and the occurrence of full-length LINE-1 transcripts in two bladder carcinoma cell lines with hypomethylated DNA (Figure 6) raises the possibility of retrotransposition occurring in urothelial carcinoma. The frequency of retrotransposition events has been found to be very high in a transfection system using retroposition-competent tagged LINE-1 elements (Moran et al, 1996) but a quantitative estimate of the frequency of retroposition events in tumour cells is not yet available. A second consequence of DNA hypomethylation of repetitive sequences could be increased mitotic recombination (Chen et al, 1998) which requires the open chromatin structure usually associated with hypomethylated DNA. In this regard, the limited number of bands seen upon hybridization of Southern blots with LINE-1 probes (cf. Figure 1) suggests a remarkable degree of sequence conservation among these elements which may facilitate homologous recombination.

In contrast to LINE-1 transcripts, HERV-K transcripts could not be detected in bladder carcinoma cell lines, but rather were restricted to male teratocarcinoma cell lines. Assuming that hypomethylation of these sequences is related to their expression, there are two potential explanations for this finding. For one, the crucial sites in the HERV-K LTR may not have become demethylated, since the most proximal site investigated (M1, located approximately 300-bp downstream from the start of transcription) was only demethylated in the cell line HT1376, but not in 5637. It remains possible therefore that demethylation of one or several of the approximately 12 CpG sites upstream of M1 is crucial for transcription of HERV-K proviruses. The situation is different with the LINE-1 promoter which comprises the CpG rich 5′-end of the elements. The first 200 bp act as the actual promoter whose function is supported by the adjacent downstream 500 bp (Swergold, 1990). The results of Southern analysis and LM-PCR indicate that sites M1–M3 located within the basal promoter and site M4 located in the supporting region are demethylated in carcinomas with DNA hypomethylation in many LINE-1 elements allowing transcription. Alternately, the LINE-1 promoter contains mainly binding sites for ubiquitous transcription factors, whereas the HERV-K LTR may be activated by cell type-specific proteins restricted to developing germ cells. Therefore, diminished methylation of these promoters may lead to activation of both kinds of promoters in teratocarcinoma cells, but of only LINE-1 promoters in somatic cells.

### ACKNOWLEDGEMENTS

This study was funded by the Deutsche Forschungsgemeinschaft (Schu604/7-1) and supported by the Centre for Biological and Medical Research at the Heinrich-Heine-Universität. We are obliged to several members of the Urology and Pathology departments for their help with tissue dissection, to André Radzewitz for technical support and to Christine Steinhoff for performing statistical analyses.

### REFERENCES

Alves G, Tatro A and Fanning T (1996) Differential methylation of human LINE-1 retrotransposons in malignant cells. *Gene* **176**: 39–44
Baylin SB, Herman JG, Graff JR, Vertino P and Issa JP (1998) Alterations in DNA methylation: a fundamental aspect of neoplasia. *Adv Cancer Res* **72**: 141–196
Bedford MT and van Helden PD (1987) Hypomethylation of DNA in pathological conditions of the human prostate. *Cancer Res* **47**: 5274–5276
Bratthauer GL and Fanning TG (1992) Active LINE-1 retrotransposons in human testicular cancer. *Oncogene* **7**: 507–510
Chen RZ, Petterson U, Beard C, Jackson-Grusby L and Jaehnisch R (1998) DNA hypomethylation leads to elevated mutation rates. *Nature* **395**: 89–93
Clifford SC, Prowse AH, Affara NA, Buys CHCM and Maher ER (1998) Inactivation of the von Hippel-Lindau (VHL) tumour suppressor gene and allelic losses at chromosome arm 3p in primary renal cell carcinoma. *Genes Chromosomes Cancer* **22**: 200–209
Dombroski BA, Scott AF and Kazazian HH (1993) Two additional potential retrotransposons isolated from a human L1 subfamily that contains an active retrotransposable element. *Proc Natl Acad Sci USA* **90**: 6513–6517
Feinberg AP, Gehrke CW, Kuo KC and Ehrlich M (1988) Reduced genomic 5-methylcytosine content in human colonic neoplasia. *Cancer Res* **48**: 1159–1161
Feng Q, Moran JV, Kazazian HH and Boeke JD (1996) Human L1 retrotransposon encodes a conserved endonuclease required for retrotransposition. *Cell* **87**: 905–916
Goelz SE, Vogelstein B, Hamilton SR and Feinberg AP (1985) Hypomethylation of DNA from benign and malignant human colon neoplasms. *Science* **228**: 187–190
Gonzalez-Zulueta M, Bender CM, Yang AS, Nguyen TT, Beart RW, van Tornout JM and Jones PA (1995) Methylation of the 5′CpG island of the p16/CDKN2 tumour suppressor gene in normal and transformed human tissues correlates with gene silencing. *Cancer Res* **55**: 4531–4535
Gotzinger N, Sauter M, Roemer K and Mueller-Lantzsch N (1996) Regulation of endogenous retrovirus-K Gag expression in teratocarcinoma cell lines and human tumours. *J Gen Virol* **77**: 2983–2990
Grimm MO, Jügens B, Schulz WA, Decken K, Makri D and Schmitz-Dräger BJ (1995) Inactivation of tumour suppressor genes and deregulation of the c-myc gene in urothelial cancer cell lines. *Urol Res* **23**: 293–300
Hata K and Sakaki Y (1997) Identification of critical CpG sites for repression of L1 transcription by DNA methylation. *Gene* **189**: 227–234
Herman JG, Latif F, Weng Y, Lerman MI, Zbar B, Liu S, Samid D, Duan DSR, Gnarra JR, Linehan WM and Baylin SB (1994) Silencing of the VHL tumor-suppressor gene by DNA methylation in renal carcinoma. *Proc Natl Acad Sci USA* **91**: 9700–9704
Herman JG, Merlo A, Mao L, Lapidus RG, Issa JPJ, Davidson NE, Sidransky D and Baylin SB (1995) Inactivation of the CDKN2/p16/MTS1 gene is frequently associated with aberrant DNA methylation in all common human cancers. *Cancer Res* **55**: 4525–4530
Holmes SE, Dombroski BA, Krebs CM, Boehm CD and Kazazian HH (1994) A new retrotransposable human L1 element from the LRE2 locus on chromosome 1q produces a chimaeric insertion. *Nat Genet* **7**: 143–148
Jarrard DF, Bova GS, Ewing CM, Pin SS, Nguyen SH, Baylin SB, Cairns P, Sidransky D, Herman JG and Isaacs WB (1997) Deletional, mutational, and methylation analyses of CDKN2 (p16/MTSI) in primary and metastatic prostate cancer. *Genes Chromosomes Cancer* **19**: 90–96
Jürgens B, Schmitz-Dräger BJ and Schulz WA (1996) Hypomethylation of L1 LINE sequences prevailing in human urothelial carcinoma. *Cancer Res* **56**: 5698–5703
Kazazian HH and Moran JV (1998) The impact of L1 retrotransposons on the human genome. *Nat Genet* **19**: 19–24
Laird PW (1996) The role of DNA methylation in cancer. *Annu Rev Genet* **30**: 441–464
Liu J, Nau MM, Zucman-Rossi J, Powell JI, Allegra CJ and Wright JJ (1997) LINE-I element insertion at the t(11;22) translocation breakpoint of a desmoplastic small round cell tumor. *Genes Chromosomes Cancer* **18**: 232–239
Löwer R, Löwer J, Tondera-Koch C and Kurth R (1993) A general method for the identification of transcribed retrovirus sequences (R-U5 PCR) reveals the expression of the human endogenous retrovirus loci HERV-H and HERV-K in teratocarcinoma cells. *Virology* **192**: 501–511
Löwer R, Löwer J and Kurth R (1996) The viruses in all of us: characteristics and biological significance of human endogenous retrovirus sequences. *Proc Natl Acad Sci USA* **93**: 5177–5184
Löwer R, Tönjes RR, Korbmacher C, Kurth R and Löwer J (1995) Identification of a rev-related protein by analysis of spliced transcripts of the human endogenous retroviruses HDTV/HERV-K. *J Virol* **69**: 141–149
Makos M, Nelkin BD, Reiter RE, Gnarra JR, Brooks J, Isaacs W, Linehan M and Baylin SB (1993) Regional DNA hypermethylation at D17.5 precedes 17p structural changes in the progression of renal tumors. *Cancer Res* **53**:

© 1999 Cancer Research Campaign

2719–2722
Merlo A, Herman JG, Mao L, Lee DJ, Gabrielson E, Burger PC, Baylin SB and Sidransky D (1995) 5′ CpG island methylation is associated with transcriptional silencing of the tumour suppressor p16/CDKN2/MTS1 in human cancers. *Nat Med* **7**: 686–692
Miki Y, Nishisho I, Horii A, Miyoshi Y, Utsunomiya J, Kinzler KW, Vogelstein B and Nakamura Y (1992) Disruption of the APC gene by a retrotransposal insertion of L1 sequence in a colon cancer. *Cancer Res* **52**: 643–645
Miller OJ, Schnedl W, Allen J and Erlanger BF (1974) 5-Methylcytosine localised in mammalian constitutive heterochromatin. *Nature* **251**: 636–637
Moran JV, Holmes SE, Naas TP, DeBerardinis RJ, Boeke JD and Kazazian HH (1996) High frequency retrotransposition in cultured mammalian cells. *Cell* **87**: 917–927
Morse B, Rothberg PG, South VJ, Spandorfer JM and Astrin SM (1988) Insertional mutagenesis of the myc locus by a LINE-1 sequence in a human breast carcinoma. *Nature* **355**: 87–90
Nagarajan L, Lange B, Cannizzaro L, Finan J, Nowell P and Huebner K (1990) Molecular anatomy of a 5q interstitial deletion. *Blood* **75**: 82–87
Pomykala HM, Bohlander SK, Broeker PL, Olopade OI and Diaz MO (1994) Breakpoint junctions of chromosome 9p deletions in two human glioma cell lines. *Mol Cell Biol* **14**: 7604–7610
Santourlidis S, Florl A, Ackermann R, Wirtz HC and Schulz WA (1999) High frequency of alterations in DNA methylation in adenocarcinoma of the prostate. *Prostate* **39**: 166–174
Schulz WA (1998) DNA methylation in urological malignancies. *Int J Oncol* **13**: 151–167
Skowronski J, Fanning TG and Singer MF (1988) Unit-length Line-1 transcripts in human teratocarcinoma cells. *Mol Cell Biol* **8**: 1385–1397
Steigerwald SD, Pfeifer GP and Riggs AD (1990) Ligation-mediated PCR improves the sensitivity of methylation analysis by restriction enzymes and detection of specific DNA strand breaks. *Nucleic Acids Res* **18**: 1435–1439
Swergold GD (1990) Identification, characterization, and cell specificity of a human LINE-1 promoter. *Mol Cell Biol* **10**: 6718–6729
Tönjes RR, Loewer R, Boller K, Denner J, Hasenmaier B, Kirsch H, Konig H, Korbmacher C, Limbach C, Lugert R, Phelps RC, Scherer J, Thelen K, Loewer J and Kurth R (1996) HERV-K: the biologically most active human endogenous retrovirus family. *J AIDS & Hum Retrovirol* **13**: 261–267
Woodcock DM, Lawler CB, Linsenmeyer ME, Doherty JP and Warren WD (1997)

*© 1999 Cancer Research Campaign*
